# Time‐dependent wound management protocol prevents periprosthetic joint infection in total knee arthroplasty with persistent drainage

**DOI:** 10.1002/ksa.70077

**Published:** 2025-09-29

**Authors:** Filippo Leggieri, Francesco Ferriani, Enrico Di Benedetto, Enrick Miani, Enrico Festa, Giovanni Balato, Stefano M. P. Rossi, Francesco Benazzo, Andrea Baldini

**Affiliations:** ^1^ Department of Clinical Orthopaedics Azienda Universitaria Ospedaliera Careggi, Università degli Studi di Firenze Firenze Italy; ^2^ Orthopedic Unit, Istituto Fiorentino di Cura e Assistenza (IFCA) Florence Italy; ^3^ SOC Ortopedia e Traumatologia, Azienda Sanitaria Universitaria Friuli Centrale Tolmezzo Italy; ^4^ Department of Public Health Orthopedic Unit, “Federico II” University Naples Italy; ^5^ Department of Life Science Health, and Health Professions, Università degli Studi Link Roma Italy; ^6^ Unità di Chirurgia Robotica UOC Ortopedia e Traumatologia, Fondazione Poliambulanza Brescia Italy; ^7^ UOC Ortopedia e Traumatolgia, Fondazione Poliambulanza Brescia Italy; ^8^ IUSS Istituto Universitario di Studi Superiori Pavia Italy

**Keywords:** DAIR (debridement, antibiotics, and implant retention), periprosthetic joint infection (PJI), persistent wound drainage, total knee arthroplasty (TKA)

## Abstract

**Purpose:**

This study aimed to evaluate the success rate of a standardised protocol for managing persistent wound drainage following total knee arthroplasty (TKA).

**Methods:**

A prospective observational study was conducted at four institutions between January and June 2022 across primary and revision TKAs. Sixty‐two patients (mean age 71.5 ± 9.3 years; 45.2% male) who experienced postoperative wound drainage after primary (58.1%) or revision (41.9%) TKA were enrolled. The protocol included staged interventions: anticoagulation adjustments and dressing changes during Days 1–3, incisional negative pressure wound therapy during Days 3–5, and wound debridement above the joint capsule if drainage persisted beyond Day 7. Outcomes were evaluated using the ASEPSIS score. The primary outcome was defined as complete wound healing without progression to debridement, antibiotics and implant retention (DAIR) procedure or periprosthetic joint infection (PJI).

**Results:**

The mean follow‐up was 13.2 months (range, 11.7–15.1 months). The protocol achieved a 91.9% success rate in preventing progression to DAIR. The median number of visits required to achieve wound healing was 3.5 for the cohort. DAIR rate was 8.1% (*n* = 5), while PJI rate was 1.6% (*n* = 1). Primary TKA procedures demonstrated more efficient healing trajectories compared to revision cases, requiring fewer clinical visits (median 3 vs. 5, *p* < 0.001) and shorter hospital stays (mean 4.5 vs. 7.5 days, *p* = 0.017). Cox proportional hazards model identified intervention type (revision vs. primary, hazard ratio [HR] 0.24, *p* < 0.001), advanced age (HR: 0.95 per year, *p* = 0.006), and male gender (HR: 0.45, *p* = 0.020) as significant independent predictors of increased time required to achieve wound healing.

**Conclusion:**

Our standardised wound management protocol achieved a high success rate in preventing progression to PJI following TKA. The time‐dependent approach to intervention escalation provides clear clinical decision‐making criteria and suggests that early identification and systematic management of wound complications are critical determinants of successful outcomes, particularly for primary TKA procedures.

**Level of Evidence:**

Level II.

**Abbreviations**
ASA: American Society of Anesthesiologists
ASEPSIS: assessment of serous discharge, purulent exudate, separation of deep tissues, isolation of bacteria and stay as inpatient prolonged
BMI: body mass index
CCS: chronic corticosteroid therapy (also referred to as systemic corticosteroid therapy)
CI: confidence interval
DAIR: debridement, antibiotics and implant retention
DMARDs: disease‐modifying antirheumatic drugs
DOACs: direct oral anticoagulants
HR: hazard ratio
iNPWT: incisional negative pressure wound therapy
LMWH: low molecular weight heparin
PJI: periprosthetic joint infection
STROBE: strengthening the reporting of observational studies in epidemiology
TKA: total knee arthroplasty

## INTRODUCTION

Wound‐related complications in primary total knee arthroplasty (TKA) can occur in approximately 0.2%–21% of the cases and include wound dehiscence, skin‐edge necrosis, superficial infection, delayed healing and persistent wound drainage [27]. Most of these complications are minor and rarely require surgical intervention, but in some cases, the sequelae can be devastating. Indeed, these conditions quadruple the risk of periprosthetic joint infection within 5 years after total knee replacement [15]. Therefore, careful long‐term monitoring is strongly recommended to avoid further complications. Among the conditions mentioned above, the most common is represented by persistent wound drainage, defined as a discharge that continues for greater than 72 h. In most cases, the drainage is sero‐haematic due to the use of anticoagulation drugs in the perioperative phase and can be classified into four stages, from limited to excessive, related to wound dressing changes needed. The continuous and prolonged fluid flow from the wound increases mechanical tension and stress on the surrounding tissues. It may provide an open barrier for skin microbiome, thus provoking an optimal environment for bacterial proliferation and a surgical site infection onset [25]. Early management of a draining wound that shows no signs of infection can be approached conservatively through local wound care, limb elevation, joint immobilisation and temporary discontinuation of anticoagulation [1]. Closed incision negative pressure wound therapy (iNPWT) implementation [8, 14] can be a valid solution to manage the discharge, promoting wound healing. However, if draining continues for more than 7 days despite the application of the nonoperative measures, irrigation and debridement associated with synovectomy and liner exchange are strongly recommended if the fascia is not intact. So, despite conservative or surgical actions to be undertaken in the presence of wound drainage being well‐described and analysed by the International Consensus Meeting on Musculoskeletal Infection [18], there is a lack in providing clear guidance on which therapeutic actions should be done depending on the days and amount of drainage. A recent survey demonstrated a lack of consensus among clinicians on the recognition and management of postoperative wound leakage, and considerable variability in practice [5]. The variability noted is consistent with a lack of formal evidence‐based guidelines, as well as the challenging nature of managing this complication.

Therefore, this study aimed to evaluate the success rate of a standardised protocol for managing persistent wound drainage following total knee replacement.

## MATERIALS AND METHODS

### Design and population

**Figure 1:**
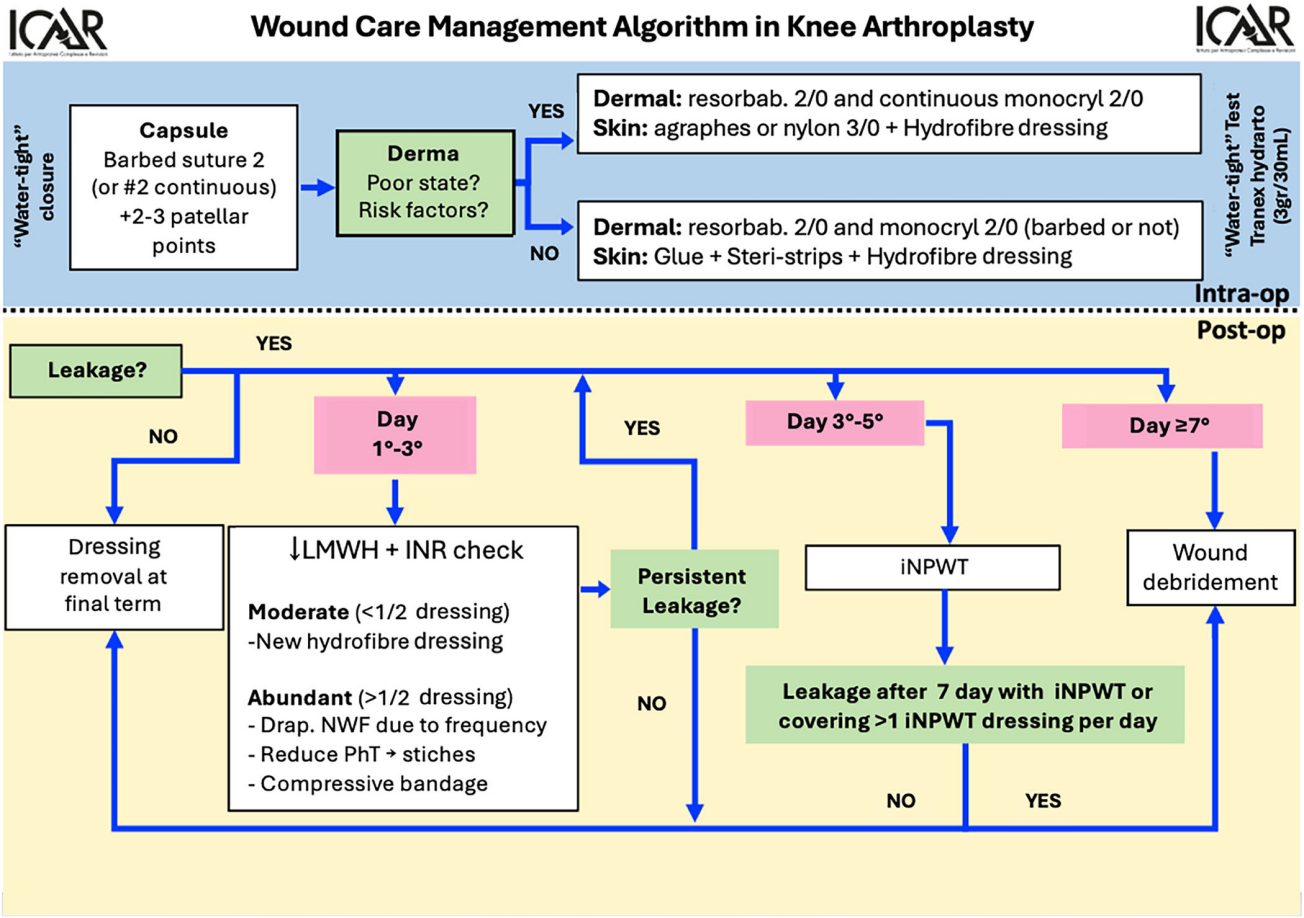
All centres implemented standardised surgical wound closure techniques and followed identical postoperative wound management protocols. The management algorithm involved closing the joint capsule with either barbed or continuous sutures, reinforced with specific resorbable patellar suture points. Water‐tight closure was verified through an intra‐articular injection of tranexamic acid (3 g/30 mL). For skin closure, a decision‐making approach was implemented based on dermal tissue quality and risk factors. In cases with poor dermal state or identified risk factors, the protocol specified dermal closure using resorbable 2/0 and continuous monocryl 2/0, with skin closure using agraphes or nylon 3/0 plus hydrofibre dressing (Aquacel®, ConvaTec Inc.). For patients without poor dermal state or risk factors, the alternative approach utilised dermal closure with resorbable 2/0 and monocryl 2/0 (either barbed or not), while skin closure was achieved using glue with Steri‐strips and hydrofibre dressing (Aquacel®, ConvaTec Inc.). Aquacel® dressings were utilised and maintained until the standard 15‐day postoperative check‐up unless signs of drainage developed earlier. Postoperatively, the management of wound secretions followed a progressive protocol. During the initial 3 days, interventions focused on anticoagulation adjustments and appropriate wound dressing applications. If drainage persisted from Days 3 to 5, incisional negative pressure wound therapy (iNPWT) was implemented. Persistent secretions beyond 7 days necessitated surgical intervention, with the approach determined by joint capsule integrity: for an intact capsule, procedures were limited to above‐capsule wound revision, while compromised capsules required more invasive DAIR (debridement, antibiotics, implant retention) surgery below the capsule.

We conducted a prospective observational study at multiple institutions between January and June 2022, evaluating the effectiveness of a standardised wound management protocol in patients undergoing TKA with postoperative wound drainage. Patient enrollment and data collection occurred between January and May 2022. The study followed the STROBE guidelines [6], and the Ethical Committee approved the trial protocol (Protocol No. 100032444/20773_oss). Patients have been enrolled in four different hospitals, which strictly followed the same watertight wound closure procedure at the time of surgery and the same postoperative protocol with the same data collection procedure in case of wound issue (Figure 1). Antibiotic prophylaxis consisting of cefazolin, or vancomycin for patients with beta‐lactam allergies, was strictly adhered to across all study centres. The same early mobilisation and anticoagulant prophylaxis protocols were strictly followed by each centre. Thromboprophylaxis was administered using either enoxaparin 4000UI or direct oral anticoagulants (DOACs), with the specific agent selected based on individual patient antithrombotic risk factors. Antihemorrhagic prophylaxis consisting of 1 g intravenous tranexamic acid was administered preoperatively at all centres, with a second 1 g dose given 4–6 h postoperatively.

Inclusion criteria were adult patients aged 18 years or older undergoing primary or revision TKA who experienced postoperative wound drainage requiring implementation of our standardised wound management protocol. Patients with evidence of infection at the time of surgery, those unable to comply with the follow‐up schedule, and those whose informed consent was not obtained were excluded. Patient eligibility was assessed daily during the postoperative period by trained research staff.

A total of 62 patients were prospectively included in the analysis with a mean age of 71.5 ± 9.3 years, of whom 28 (45.2%) were male. Primary TKA was performed in 36 patients (58.1%), while 26 (41.9%) underwent revision TKA. Comorbidities included diabetes in a subset of patients, smoking, and varying BMI classifications. The baseline characteristics of the study population are summarised in Table 1.

**Table 1:** Overall baseline characteristics.

Variable	Values
BMI > 30 kg/m² [*n*(%)]	31 (50.0%)
Primary TKA [*n*(%)]	36 (58.1%)
Pre‐op anticoagulation [*n*(%)]	14 (22.6%)
Hypertension [*n*(%)]	35 (56.5%)
Diabetes mellitus [*n*(%)]	9 (14.5%)
Active smoking [*n*(%)]	8 (12.9%)
Pulmonary conditions [*n*(%)]	5 (8.1%)
Vascular diseases [*n*(%)]	4 (6.5%)
Ongoing corticosteroid therapy [*n*(%)]	6 (9.7%)
DMARDs use [*n*(%)]	2 (3.2%)
Rheumatic pathologies [*n*(%)]	7 (11.3%)
Previous infections [*n*(%)]	9 (14.5%)
Dermatological conditions [*n*(%)]	5 (8.1%)
No comorbidities [*n*(%)]	11 (17.7%)
1‐2 comorbidities [*n*(%)]	38 (61.3%)
3‐4 comorbidities [*n*(%)]	13 (21.0%)
ASA ≥ 3 [*n*(%)]	20 (32.3%)
Multiple previous incisions [n(%)]	19 (30.6%)

Abbreviations: ASA, American Society of Anesthesiologists; BMI, body mass index; DMARDs, disease‐modifying antirheumatic drugs; TKA, total knee arthroplasty.

### Primary and secondary outcomes

The primary outcome was defined as complete wound healing without progression to debridement, antibiotics and implant retention procedure (DAIR) or PJI. Success was specifically defined as achieving complete wound healing characterised by: ASEPSIS score of 0, indicating absence of drainage, erythema, or wound complications; no requirement for additional outpatient visits; and absence of DAIR procedure. Wound debridement was not classified as treatment failure, but rather as an integrated step within the established protocol.

The distinction between DAIR and wound debridement was determined intraoperatively based on capsular integrity. After thorough debridement of supracapsular tissues, if the joint capsule remained intact, the procedure was considered complete and classified as wound debridement within the drainage management protocol (Figure 1). Conversely, if the capsule was compromised, the procedure progressed to formal DAIR as the skin breach extending beyond the capsule represented a continuity with the external knee environment, with a significantly increased risk for additional deep infection surgery. Intraoperative tissues sampling for microbiological evaluation were obtained to target antimicrobial treatment.

In cases where the capsule remained intact and in the absence of systemic and local signs of infection, PJI diagnosis was not established. However, synovial fluid was routinely collected for microbiological analysis and white blood cell (WBC) count and Polymorphonuclear (PMN) percentage to confirm the absence of infection or identify potential pathogens subsequently. If microbiological results returned positive, patients were returned to the operating room for formal DAIR. This strategy was based on the rationale that in cases of an intact capsule with evidence of superficial wound and soft tissue infection at the same time, opening a virtually intact capsule could increase the risk of iatrogenic infection.

Any surgical revisions that addressed only the wound without entering the joint capsule were not classified as DAIR procedures. Instead, these less invasive wound revisions were categorised as expected interventions within our standard treatment protocol (Figure 1).

PJI was diagnosed according to the International Consensus Meeting on Musculoskeletal Infection diagnostic criteria [18].

### Data collection and follow‐up

The following data were collected for each patient: baseline characteristics, comorbidities such as diabetes mellitus, active smoking status, obesity (defined as BMI > 30 kg/m²), history of previous incisions in the surgical area, ongoing corticosteroid therapy, use of immunomodulators, rheumatic pathologies and the anticoagulation regimen needed before surgery.

Throughout the postoperative period, wound characteristics were systematically evaluated at each dressing change through the ASEPSIS score [29]. The ASEPSIS score is a validated clinical tool that quantifies postsurgical wound healing by combining visual wound characteristics with clinical outcomes, meaning the assessment of serous or haematic drainage, presence of purulent exudate, peri‐incisional erythema and wound dehiscence. It provides an objective measure, with higher scores indicating more severe healing disturbances [29]. Patients with ASEPSIS scores ≤10 were classified as low complexity, those with scores >10 and ≤20 as medium complexity, and those with scores >20 as high complexity [30].

Wound evaluations occurred daily throughout the hospital stay and at the scheduled 15‐day postoperative outpatient visit. Following discharge, additional outpatient assessments occurred for patients who sustained significant dressing saturation (>1/3 capacity) or evidence of wound leakage. All interventions performed during these dressing changes were recorded. Complications of the surgical site occurring within 60 days postsurgery, and any other complication during the follow‐up period were recorded. Any evidence of periprosthetic joint infections within the first year postoperatively were also recorded. Any need for surgical reintervention was recorded, including the date, cause and specific actions taken.

### Sample size calculation

For our primary outcome of preventing progression to PJI in patients with wound leakage, we performed a sample size calculation. Based on published literature showing PJI rates ranging up to 50% following persistent wound drainage [7, 19, 23] and consistent with the largest population from LEAK protocol assumption of 20% [13], we estimated a 20% infection rate in untreated patients. We hypothesised our protocol would reduce this to 4%. With *α* = 0.05 and 80% power, this yielded a minimum required sample of 60 patients, which matched our final cohort size. This assumption was based on the rationale that early, systematic intervention could achieve PJI rates approaching those observed in uncomplicated primary arthroplasty (0.6%–1.3% [24]) while accounting for the inherently elevated risk associated with persistent wound drainage. The targeted reduction reflects our clinical hypothesis that proactive, time‐dependent management could prevent the majority of drainage‐related infections through early intervention before bacterial colonisation and deep tissue involvement occur. Moreover, the published literature demonstrates highly variable PJI rates following persistent drainage, ranging from 1.3% to 50% [7, 19, 23], indicating substantial room for improvement with the current systematic intervention.

### Data analyses

Continuous variables were analysed using the Mann–Whitney *U* test, while categorical variables were expressed as proportions and compared using the Fisher exact or the chi‐square test, as appropriate. Time to complete wound healing was analysed using Kaplan–Meier survival analysis, with log‐rank tests to compare healing times between subgroups. Cox proportional hazards regression was used to identify risk factors for delayed healing while adjusting for potential confounders. A *p*‐value of <0.05 indicated significance. R software (R Core Team, R Foundation for Statistical Computing) and IBM SPSS 25.0 software (IBM Corp) were used to conduct the statistical analyses.

### Ethical aspects and intercentre standardisation

The study was conducted in accordance with the Declaration of Helsinki following approval by the Institutional Ethics Committee of Giomi Innovation and Research No. 100032444/20773_oss. All participants provided written informed consent prior to study enrollment. Initial training across centres was conducted during prestudy protocol development meetings, where all procedures were carefully and collaboratively defined. Two additional consensus meetings were subsequently held via teleconference to ensure standardisation of closure techniques, algorithm adherence and wound revision protocols across all participating centres.

## RESULTS

The mean time between surgery to discharge was 5.8 ± 3.42 days (range, 2–21). The mean follow‐up was 13.2 months (range, 11.7–15.1 months). The interventions to the wound included hydrofiber dressings (*n* = 87) (Aquacel, Ag Surgical, Convatec Inc.), compression bandaging (*n* = 59), nonwoven fabric dressings (*n* = 54), physical therapy adjustments (*n* = 37), suture correction (*n* = 31), incisional negative pressure wound therapy (iNPWT—Avelle®, Convatec Inc.) (*n* = 22), low molecular weight heparin (LMWH) reduction (*n* = 10) and surgical wound revision (*n* = 1). Of the total postoperative visits conducted, a maximum of five were recorded during hospitalisation, while a maximum of three additional subsequent visits were performed in the outpatient setting after discharge, allowing for comprehensive wound monitoring throughout the healing process.

The overall protocol success rate, defined as wound healing without requiring reoperation (DAIR procedure), was 91.9% (95% confidence interval [CI]: 85.1%–98.7%, *n* = 57) The median number of visits required to achieve wound healing (ASEPSIS score of 0) was 3.5 for the entire cohort (mean 3.7 ± 1.6). DAIR rate was 8.1% (95% CI: 1.3%–14.9%) (*n* = 5), while PJI rate was 1.6% (95% CI: 0.53%–8.3%, *n* = 1). The time between surgery and DAIR for each case was 9.8 days (range, 8–13).

### Subgroup analyses

**Figure 2:**
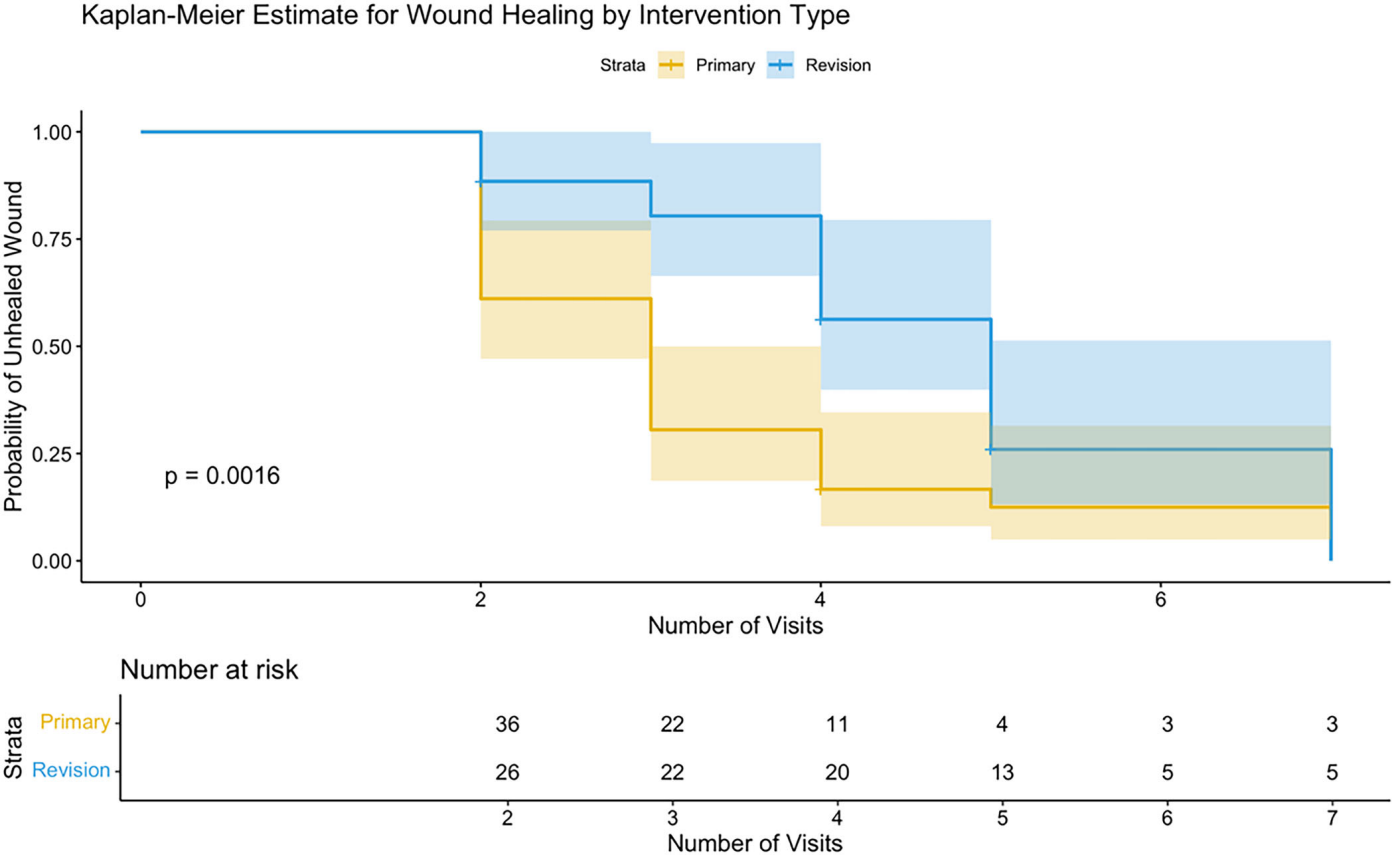
Kaplan–Meier analysis demonstrated a significant difference between primary and revision interventions in healing trajectories.

Patient demographics and wound characteristics were similar between primary and revision groups, as shown in Table 2. Given the limited sample sizes (36 primary vs. 26 revision cases), comparisons between these subgroups should be interpreted with caution due to insufficient statistical power. Primary interventions demonstrated a numerically higher success rate (94.4%, 95% CI: 81.3%–99.3%) compared to revision procedures (88.5%, 95% CI: 69.8%–97.6%), though this difference did not reach statistical significance (*p* > 0.05). Kaplan–Meier analysis suggested distinct healing patterns between groups (*p* < 0.001), with primary procedures appearing to follow a more favourable trajectory (Figure 2). Primary procedures required a median of 3 visits to healing (95% CI: 2–3), while revision procedures necessitated a median of 5 visits (95% CI: 4–NA). This disparity was reflected in the total number of visits, with primary interventions requiring a mean of 3.1 ± 1.6 visits compared to 4.3 ± 1.3 visits for revision procedures (*p* < 0.01). The DAIR rate was 5.6% for primary cases versus 11.5% for revisions, though this difference did not reach statistical significance (*p* > 0.05). However, as confirmed in the multivariable analysis, intervention type was significantly associated with healing trajectory (Table 3). Revision patients stayed significantly longer in the hospital (mean 7.5 vs. 4.5 days, *p* = 0.017, 95% CI: −5.57 to −0.56).

**Table 2:** Summary of baselines' differences between primary and revision group.

Variable	Primary	Revision	Mean difference	95% CI	*p* value
Age (years)	72.6 ± 8.5 (*n* = 36)	69.8 ± 10.4 (*n* = 26)	2.8	−2.6 to 8.4	0.302
ASEPSIS	2.7 ± 1.5 (*n* = 36)	2.7 ± 1.3 (*n* = 26)	−0.0	−0.7 to 0.7	0.981
Diabetes	2 (5.6%)	5 (19.2%)	3.9	0.5–45.1	0.119
Smoke	3 (8.3%)	2 (7.7%)	0.9	0.07–8.6	1.000
BMI (kg/m)	14 (38.9%)	6 (23.1%)	0.4	0.1–1.6	0.272
Previous skin incisions/scar	3 (8.3%)	6 (23.1%)	3.2	0.6–22.2	0.148
CCS	3 (8.3%)	1 (3.8%)	0.4	0.01–5.9	0.633
Rheumatic	4 (11.1%)	2 (7.7%)	0.6	0.06–5.1	1.000
Outpatient readmission	5 (13.9%)	1 (3.8%)	0.2	0.01–2.4	0.387
Late discharged	3 (8.3%)	2 (7.7%)	0.9	0.0–8.6	1.000
DAIR	2 (5.6%)	3 (11.5%)	2.1	0.2–28.1	0.641

Abbreviations: ASEPSIS, assessment of serous discharge, purulent exudate, separation of deep tissues, isolation of bacteria, and stay as inpatient prolonged; BMI, body mass index; CCS, systemic corticosteroid therapy; CI, confidence interval; DAIR, debridement, antibiotics and implant retention; reumathic, rheumatic pathologies.

**Table 3:** Cox proportional hazards model for factors associated with wound healing.

	Hazard ratio	95% confidence interval	*p* value
Revision vs. primary	0.2	0.1–0.5	<0.001
Age (per year)	0.9	0.9–0.9	0.006
Gender (male vs. female)	0.4	0.2–0.8	0.020
Comorbidity count	1.5	0.7–3.2	0.258

### Multivariate analysis of factors affecting healing

**Figure 3:**
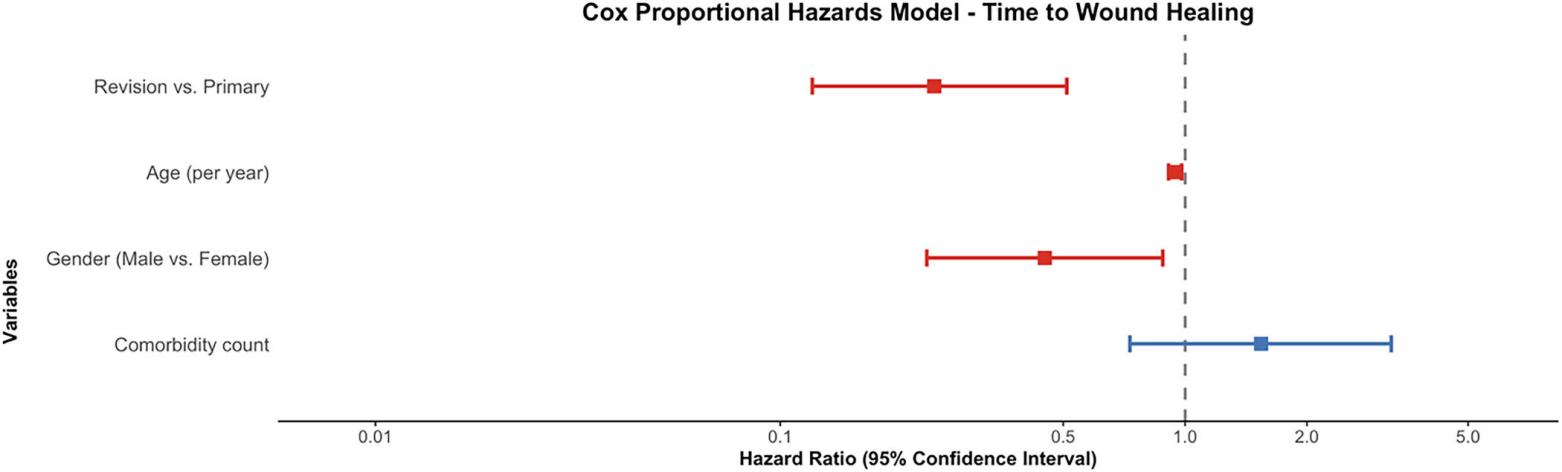
Forest plot visually describing Hazard Ratio associated with time to complete wound healing.

Cox proportional hazards model identified several significant independent predictors of wound healing efficiency, as measured by the number of visits required to achieve an ASEPSIS score of 0. The overall model demonstrated good discriminative ability with a concordance statistic of 0.786 (SE = 0.036) and was highly significant according to likelihood ratio test (*χ*² = 22.07, *df* = 6, *p* = 0.001), Wald test (*χ*² = 19.84, *df* = 6, *p* = 0.003) and logrank test (*χ*² = 22.28, *df* = 6, *p* = 0.001) (Table 3). Intervention type, age and gender were the most robust predictors of wound healing efficiency in the cohort (Figure 3).

## DISCUSSION

The most important finding of this study was that our standardised wound management protocol achieved a high success rate of 91.9% (with 8.1% DAIR rate) in managing persistent wound drainage following TKA, effectively preventing progression to periprosthetic joint infection in most cases.

Notably, while our revision cases showed a slightly higher DAIR rate compared to primary TKAs (11.5% vs. 5.6% in primary TKA), this difference did not reach statistical significance. Most importantly, despite these reoperations, the incidence of confirmed PJI was remarkably low at 1.6% (*n* = 1) in a population specifically selected for having wound leakage. This compares favourably to both the general epidemiological PJI incidence after primary arthroplasty (0.6%–1.3% [24]) and the much higher PJI rates associated with persistent wound drainage (1.3%–50% [7, 19, 23]), suggesting that our protocol effectively mitigated the increased infection risk typically associated with persistent drainage.

The timing of interventions in our protocol deserves particular emphasis. Current evidence suggests that when persistent wound drainage continues beyond 5–7 days following the initial procedure, despite appropriate nonsurgical interventions, surgical management becomes necessary [10, 17, 22, 26].

Patel et al. [19] demonstrated that each additional day of persistent wound drainage carried a 42% increased risk of wound infection in TKA. Similarly, Jaberi et al. [10] found that early surgical exploration and debridement within the first 5–7 postoperative days resolves persistent wound drainage in approximately 76% of cases, while delaying intervention may significantly increase PJI risk. Our protocol's clear timeframes for escalating care—anticoagulation adjustment and dressing changes during Days 1–3, iNPWT implementation during Days 3–5, and surgical intervention if drainage persists beyond Day 7—provided clinicians with objective criteria for decision‐making, eliminating unnecessary delays in treatment escalation.

The effectiveness of our protocol is supported by Wagenaar et al. [26], whose study advocated for early intervention in cases of persistent drainage, with the majority of surgeons implementing advanced therapeutic measures between Days 3–5 and proceeding to surgical revision when drainage continues beyond 5–7 days.

Similarly, Scuderi [22] emphasises that when a wound has been deemed problematic, at minimum, superficial exploration with debridement and haematoma evacuation should be performed promptly to prevent deeper infection.

Our findings revealed notable differences in healing trajectories between primary and revision TKA cases with wound discharge. While both groups showed comparable ultimate success rates, primary TKA patients achieved complete wound healing with fewer clinical visits (median 3 vs. 5) and shorter hospital stays (mean 4.5 vs. 7.5 days) compared to revision cases. This finding suggests that the standardised wound management protocol is effective for both primary and revision procedures, though the latter require more intensive monitoring and treatment.

The multivariate analysis identified several independent predictors of wound healing efficiency. Intervention type (primary vs. revision) emerged as the strongest determinant, with revision procedures demonstrating significantly slower healing trajectories. Additionally, advanced age and male gender were associated with prolonged healing time. While previous literature extensively documents surgical risk factors such as previous surgery, surgical approach, tourniquet use and operative time [2, 3, 4, 9, 11, 16, 19, 20, 21, 22, 28, 31, 32], our findings suggest patient‐specific characteristics may play an important role in healing trajectories. This difference may reflect the distinct focus of our study on healing efficiency within a standardised protocol rather than risk factor assessment. Our analysis specifically examined factors affecting the number of visits required to achieve complete wound healing, whereas previous studies often concentrated on the binary outcome of complication development.

Importantly, initial wound complexity as measured by the ASEPSIS score was not significantly associated with protocol failure or need for reoperation. Even patients presenting with moderate complexity wounds demonstrated comparable success rates to those with simpler wounds (92.9% vs. 91.5%), suggesting that our protocol is robust across varying degrees of initial wound compromise. This finding is particularly valuable as it indicates that early identification and systematic management of wound complications, rather than the initial severity, may be the critical determinant of ultimate outcome.

While the literature documents highly variable DAIR success rates ranging from 17% to 100% [12], reflecting differences in inclusion criteria, pathogens and treatment protocols, our 91.9% success rate in preventing progression to PJI demonstrates the value of a standardised, proactive approach to wound management. The implementation of our structured wound management protocol may have effectively controlled for many procedural variables, allowing patient‐specific factors to emerge more prominently in our analysis.

The relative homogeneity in success rates across different comorbidity profiles suggests that our protocol may be effective even in higher‐risk populations. However, it should be noted that our multivariate analysis did not identify diabetes, smoking, or obesity as independent predictors of healing efficiency, which contrasts with some previous studies [2, 3, 4, 9, 11, 16, 19, 20, 21, 22, 28, 31, 32]. This discrepancy may be attributable to the relatively small sample size, particularly for specific comorbidity subgroups, or to our analytical approach of using aggregated comorbidity count rather than entering individual comorbidities as separate categorical variables in the statistical model. While this approach preserved statistical power by avoiding model over‐parameterisation given our sample size, it may have masked individual comorbidity effects that could be detected in larger studies with sufficient power to analyse each comorbidity separately.

Several limitations of this study warrant acknowledgement. The relatively small sample size (*n* = 62) limits the precision of certain subgroup analyses and comparisons, particularly for specific comorbidity profiles. However, this sample size reflects the inherent infrequency of persistent wound drainage requiring protocol intervention in the arthroplasty population and was determined through formal power analysis to ensure adequate statistical significance for our primary outcomes. The multicentre design, while enhancing generalisability, introduces potential variability in data collection and wound assessment despite standardised training protocols across participating centres. The absence of formal calibration sessions for ASEPSIS scoring represents a methodological limitation, though post hoc analysis demonstrated acceptable intercentre consistency in intervention frequency. Finally, the prospective observational design, while appropriate for evaluating protocol effectiveness, does not provide the same level of evidence as a randomised controlled trial comparing different management strategies.

Despite these limitations, our findings provide valuable evidence supporting a standardised approach to managing persistent wound drainage following TKA. The high success rate, particularly in preventing progression to PJI, highlights the importance of early recognition, systematic evaluation and protocol‐driven intervention for postoperative wound complications. Future research should focus on refining risk stratification models to identify patients who might benefit from prophylactic implementation of advanced wound management strategies, potentially including preemptive application of iNPWT in high‐risk cases.

## CONCLUSION

Our standardised wound management protocol achieved a 91.9% success rate in preventing progression to periprosthetic joint infection following TKA, with primary procedures demonstrating more efficient healing trajectories than revision cases. The protocol's time‐dependent approach to intervention escalation provides clear temporal decision‐making criteria and suggests that early identification and systematic management of wound complications are critical determinants of successful outcomes.

## AUTHOR CONTRIBUTIONS

Andrea Baldini contributed to the study conception and design. Material preparation and data collection were coordinated by Francesco Ferriani and performed by Enrico Di Benedetto, Enrick Miani, Enrico Festa and Stefano M. P. Rossi. Statistical analyses were performed by Filippo Leggieri. Filippo Leggieri and Andrea Baldini assessed the interpretation of the data. Andrea Baldini, Giovanni Balato and Francesco Benazzo supervised the study from inception. The first draft of the manuscript was written by Filippo Leggieri. Andrea Baldini and Giovanni Balato reviewed the draft and all authors commented on previous versions of the manuscript. All authors read and approved the final manuscript.

## CONFLICT OF INTEREST STATEMENT

The authors declare no conflicts of interest.

## ETHICS STATEMENT

Approved by the Institutional Ethics Committee of Giomi Innovation and Research No. 100032444/20773_oss. The study adhered to the principles outlined in the Declaration of Helsinki. Informed consent was acquired for all the included population.

## Data Availability

The dataset is available on request.

## References

[1] Amin NH , Speirs JN , Simmons MJ , Lermen OZ , Cushner FD , Scuderi GR . Total knee arthroplasty wound complication treatment algorithm: current soft tissue coverage options. J Arthroplasty. 2019;34(4):735–742. 30665832 10.1016/j.arth.2018.12.016

[2] Ayoub F , Quirke M , Conroy R , Hill A . Chlorhexidine‐alcohol versus povidone‐iodine for pre‐operative skin preparation: a systematic review and meta‐analysis. Int J Surg Open. 2015;1:41–46.

[3] Butt U , Ahmad R , Aspros D , Bannister G . Factors affecting wound ooze in total knee replacement. Ann R Coll Surg England. 2011;93(1):54–56. 20836920 10.1308/003588410X12771863937124 PMC3293273

[4] Carroll K , Dowsey M , Choong P , Peel T . Risk factors for superficial wound complications in hip and knee arthroplasty. Clin Microbiol Infect. 2014;20(2):130–135. 23573834 10.1111/1469-0691.12209

[5] Choi M , Wheelton A , Naylor T . Management of persistent postoperative wound leakage after total hip and knee arthroplasty: a regional perspective in the north west of England. Ann R Coll Surg England. 2025 Apr 3. 10.1308/rcsann.2025.0002 PMC12578582 40178363

[6] Cuschieri S . The STROBE guidelines. Saudi J Anaesth. 2019;13(Suppl 1):31. 10.4103/sja.SJA_543_18 PMC6398292 30930717

[7] Eveillard M , Mertl P , Canarelli B , Lavenne J , Fave MH , Eb F , et al. [Risk of deep infection in first‐intention total hip replacement. Evaluation concerning a continuous series of 790 cases]. Presse Medicale. 2001;30(38):1868–1875. 11791394

[8] Gusho C , Hoskins W , Ghanem E . A comparison of incisional dressings and negative‐pressure wound therapy for the prevention of infection and wound complications after primary total hip and knee arthroplasty: a network meta‐analysis of randomized controlled trials. JBJS Rev. 2024;12(9). 10.2106/JBJS.RVW.24.00115 39283964

[9] Illingworth KD , Mihalko WM , Parvizi J , Sculco T , McArthur B , el Bitar Y , et al. How to minimize infection and thereby maximize patient outcomes in total joint arthroplasty: a multicenter approach. J Bone Jt Surg. 2013;95(8):e50. 10.2106/JBJS.L.00596 23595076

[10] Jaberi FM , Parvizi J , Haytmanek TC , Joshi A , Purtill J . Procrastination of wound drainage and malnutrition affect the outcome of joint arthroplasty. Clin Orthop Rel Res. 2008;466(6):1368–1371. 10.1007/s11999-008-0214-7 PMC2384013 18404297

[11] Jahng KH , Bas MA , Rodriguez JA , Cooper HJ . Risk factors for wound complications after direct anterior approach hip arthroplasty. J Arthroplasty. 2016;31(11):2583–2587. 27267230 10.1016/j.arth.2016.04.030

[12] Kristensen NK , Callary SA , Nelson R , Harries D , Lorimer M , Smith P , et al. Outcomes of debridement, antibiotics, and implant retention in the management of infected total knee arthroplasty: analysis of 5,178 cases from the National Australian Registry. J Arthroplasty. 2025;40:1852–1859.e1. 39710215 10.1016/j.arth.2024.12.016

[13] Löwik CAM , Wagenaar F‐C , van der Weegen W , Poolman RW , Nelissen RGHH , Bulstra SK , et al. LEAK study: design of a nationwide randomised controlled trial to find the best way to treat wound leakage after primary hip and knee arthroplasty. BMJ Open. 2017;7(12):e018673. 10.1136/bmjopen-2017-018673 PMC5770823 29288184

[14] Morgan T , Page T . The effectiveness of prophylactic closed incision negative pressure wound therapy compared to conventional dressings in the prevention of periprosthetic joint infection post hip and knee revision arthroplasty surgery: a systematic review. Int J Orthop Trauma Nurs. 2024;53:101048. 10.1016/j.ijotn.2023.101048 37845090

[15] Mortazavi JSM , Schwartzenberger J , Austin MS , Purtill JJ , Parvizi J . Revision total knee arthroplasty infection: incidence and predictors. Clin Orthop Rel Res. 2010;468(8):2052–2059. 10.1007/s11999-010-1308-6 PMC2895829 20309657

[16] Nowak LL , Schemitsch EH . Duration of surgery affects the risk of complications following total hip arthroplasty. Bone Jt J. 2019;101–B(6_Supple_B):51–56. 10.1302/0301-620X.101B6.BJJ-2018-1400.R1 31146572

[17] Parvizi J , Gehrke T , Chen AF . Proceedings of the international consensus on periprosthetic joint infection. Bone Jt J. 2013;95–B(11):1450–1452. 10.1302/0301-620X.95B11.33135 24151261

[18] Parvizi J , Tan TL , Goswami K , Higuera C , Della Valle C , Chen AF , et al. The 2018 definition of periprosthetic hip and knee infection: an evidence‐based and validated criteria. J Arthroplasty. 2018;33(5):1309–1314.e2. 29551303 10.1016/j.arth.2018.02.078

[19] Patel VP , Walsh M , Sehgal B , Preston C , DeWal H , Di Cesare PE . Factors associated with prolonged wound drainage after primary total hip and knee arthroplasty. J Bone Jt Surg Am Vol. 2007;89(1):33–38. 10.2106/JBJS.F.00163 17200307

[20] Purcell RL , Parks NL , Gargiulo JM , Hamilton WG . Severely obese patients have a higher risk of infection after direct anterior approach total hip arthroplasty. J Arthroplasty. 2016;31(9 Suppl):162–165. 10.1016/j.arth.2016.03.037 27133929

[21] Rama KRBS , Apsingi S , Poovali S , Jetti A . Timing of tourniquet release in knee arthroplasty. Meta‐analysis of randomized, controlled trials. J Bone Jt Surg Am Vol. 2007;89(4):699–705. 10.2106/JBJS.F.00497 17403789

[22] Scuderi GR . Avoiding postoperative wound complications in total joint arthroplasty. J Arthroplasty. 2018;33(10):3109–3112. 29475573 10.1016/j.arth.2018.01.025

[23] Shahi A , Boe R , Bullock M , Hoedt C , Fayyad A , Miller L , et al. The risk factors and an evidence‐based protocol for the management of persistent wound drainage after total hip and knee arthroplasty. Arthroplast Today. 2019;5(3):329–333. 31516977 10.1016/j.artd.2019.05.003 PMC6728765

[24] Springer BD , Cahue S , Etkin CD , Lewallen DG , McGrory BJ . Infection burden in total hip and knee arthroplasties: an international registry‐based perspective. Arthroplast Today. 2017;3(2):137–140. 28695187 10.1016/j.artd.2017.05.003 PMC5485227

[25] Vince K , Chivas D , Droll KP . Wound complications after total knee arthroplasty. J Arthroplasty. 2007;22(4 Suppl 1):39–44. 10.1016/j.arth.2007.03.014 17570276

[26] Wagenaar F‐C , Löwik CAM , Stevens M , Bulstra SK , Pronk Y , van den Akker‐Scheek I , et al. Managing persistent wound leakage after total knee and hip arthroplasty. Results of a nationwide survey among Dutch orthopaedic surgeons. J Bone Jt Infect. 2017;2(4):202–207. 29188171 10.7150/jbji.22327 PMC5704001

[27] Wagenaar F‐CBM , Löwik CAM , Zahar A , Jutte PC , Gehrke T , Parvizi J . Persistent wound drainage after total joint arthroplasty: a narrative review. J Arthroplasty. 2019;34(1):175–182. 30245124 10.1016/j.arth.2018.08.034

[28] Watts CD , Houdek MT , Wagner ER , Sculco PK , Chalmers BP , Taunton MJ . High risk of wound complications following direct anterior total hip arthroplasty in obese patients. J Arthroplasty. 2015;30(12):2296–2298. 26145189 10.1016/j.arth.2015.06.016

[29] Wilson APR , Sturridge MF , Treasure T , Grüneberg RN . A scoring method (ASEPSIS) for postoperative wound infections for use in clinical trials of antibiotic prophylaxis. Lancet. 1986;327(8476):311–312. 10.1016/s0140-6736(86)90838-x 2868173

[30] Wilson APR , Weavill C , Burridge J , Kelsey MC . The use of the wound scoring method ‘ASEPSIS’ in postoperative wound surveillance. J Hosp Infect. 1990;16(4):297–309. 1980502 10.1016/0195-6701(90)90002-6

[31] Wood JJ , Bevis PM , Bannister GC . Wound oozing after total hip arthroplasty. Ann R Coll Surg England. 2007;89(2):140–142. 17346407 10.1308/003588407X155509 PMC1964560

[32] Woolson ST , Mow CS , Syquia JF , Lannin JV , Schurman DJ . Comparison of primary total hip replacements performed with a standard incision or a mini‐incision. J Bone Jt Surg Am Vol. 2004;86(7):1353–1358. 10.2106/00004623-200407000-00001 15252080

